# Dyspnœa in Heart Disease

**Published:** 1910-02-05

**Authors:** 

**Affiliations:** Physician to the Laennec Hospital, Paris


### Hospital Clinics.

### DYSPNŒA IN HEART DISEASE.

By DR. BARRIE, Physician to the Laennec Hospital, Paris.

(From Notes on a Clinical Lecture.)

Dyspnœa, as everybody knows, is one of the prime manifestations of heart disease, and, in fact, the one patients most frequently complain of. Its causes are numerous, and differ according to the stage of the disease. The prognosis, also, varies greatly. In the first stages of organic disease of the heart dyspnœa is generally very slight; it is transient, coming on only after sudden or violent exertion. Later on, when compensation and the circulatory equilibrium no longer exist, it is brought on by the slightest exertion; finally, in the period of asystolism, it forms one of the leading symptoms of the disease, and the one which causes the most distress to the patients.

We thus see that—contrary to what is observed in diseases of other organs—the earliest and most important symptom in heart disease is not a cardiac one, but a respiratory one. This point, paradoxical as it may seem at first sight, is explained by the fact that the lung is interposed between the right and left sides of the heart, and is the first organ to support the consequences of any cardiac perturbation. It is this variety of dyspnœa, which is known since Corvisart's time under the name of "*dyspnée d'effort*"; it is the first symptom to appear in cardiopathics, and is that which first calls the physician's attention to the heart.

### A Classical Description.

Corvisart, Napoleon's famous physician, described it as follows:—"In patients suffering from heart disease the slightest exertion gives rise to intense breathlessness; every now and then the patient, in order to breathe more easily, is compelled to stop walking, especially if he happens to be going upstairs. With the progress of the disease respiratory trouble increases, and there is, it may be, a slight but permanent difficulty in breathing; and, whenever the patient tries to walk quickly he has to stop, for breathing becomes impossible. The same thing occurs if he has a fatiguing profession, or if he tries walking up hill or climbing stairs." Dyspnœa is also brought on by sudden or violent emotion—in a word, by all acts, psychical or muscular, which increase the frequency of the heart beat. It is determined, in fine, by the increase of arterial pressure caused by exertion, which demands from the heart a greater effort than, in its diseased state, it is capable of giving.

Exertion presents another cause of fatigue for the heart, for, as Marey has shown, in the physiological state, the heart-beat is more rapid during expiration than inspiration, and therefore exertion, necessitating an intense effort of expiration with the glottis closed, increases its frequency.

For the same reason muscular exercise is a cause of fatigue for the heart. Bryan-Robinson, in a man who had 64 pulsations per minute at rest and 78 after walking slowly, saw them go up to 140, 150, and more, after running quickly. Simply changing one's position is sufficient to accelerate the heart and pulse. Guy, observing the pulse, noted an average of 66 pulsations per minute in men lying down, 71 as soon as they sat up, and 81 when standing up.

### Varieties of Dyspnœa.

When the heart affection is fully developed dyspnœa may be due to several causes, and, from the clinical point of view, may be distinguished into three varieties: *Mechanical D*, *toxic D*, and *reflex D* (or dyspnœa having a nervous origin). Habitually mechanical dyspnœa is the consequence of chronic pulmonary congestion, commonly designated as passive congestion, which comes on even in the period of hyposystolism, but especially in that of asystolism. Its pathogeny is exceedingly simple, and, taking a patient presenting a mitral affection as example, may be described as follows: Owing to the existence of lesions the left auricle is emptied imperfectly, pressure in the pulmonary veins is increased, and the blood-stream is slowed in the lung. Very soon œdematous congestion takes place, particularly at the base of the lungs and most frequently on the side on which the patient is in the habit of lying. This passive congestion develops slowly and insidiously, gives rise to increased dyspnœa, cough, and pinkish sero-mucous expectoration. Slight dulness at the bases, obscurity of the respiratory murmur, sibilant and sonorous râles, and especially fine mucous râles, are the physical signs found on examination.

Instead of being chronic and passive the congestion of the lung may be acute, and two varieties may be described. The first is benign, and consists in hyperæmia due to increased activity of the left heart: Lasègne has given a good description of this form. It is more frequently noted in aortic lesions, passive congestion being more frequent in mitral disease. Active hyperæmia comes on in attacks which both appear and disappear rapidly; the patient presents marked oppression, pinkish expectoration, and sometimes there is even slight hæmoptysis. On examination, one finds fine mucous râles especially at the bases, but also in the axillary regions and sometimes in the apices. In the second variety, studied by Andral, Fournet, Grisolle, and more recently by Huchard and Bouveret, three clinical forms have been noted.

The *first* runs a very rapid course, and is characterised by actual bronchial paralysis or bronchoplegia. All expectoration is suppressed, and the patient dies rapidly, choked by the accumulation of frothy serum which obliterates the bronchial tubes. The *second* begins like an attack of asthma. Very soon the dyspnœa becomes orthopnœa, the patient is compelled to sit up in his bed, he is covered with perspiration, and presents either cyanosis or extreme pallor. Breathing is very laboured; there is cough, and he brings up sero-albuminous and frothy sputum, generally white but sometimes pinkish, and streaked with a little blood. At first the heart pulsations are regular and rapid, but they soon become weak and irregular. On auscultation, one finds fine mucous râles disseminated throughout both lungs. The dyspnœa becomes more and more intense, cyanosis increases and the patient dies in the space of a few hours. The post-mortem shows the lungs to be increased in volume, of a pinkish or pale greyish colour, and giving scarcely any crepitation to pressure with the finger. On section one finds "an absolute œdematous inundation" of the organ and a large quantity of frothy serum runs off. In the *third* form the symptoms come on less rapidly and less suddenly; still, however, they most frequently end fatally, death coming on during an attack of asystolism.

### Dyspnœa in Arterio-Sclerotic Patients.

We have just seen that acute congestive œdema of the lungs resembles to a certain extent an attack of asthma or of dyspnœic uræmia. The latter may be distinguished by the presence of the ordinary symptoms of chronic nephritis, polyuria, pollakiuria, albuminuria, "bruit de galop," etc. Asthma, on the other hand, by its tendency to periodicity, its nocturnal paroxysms, and the good state of the general health during the intervals of the attacks. I will not insist any further on the pathogeny of acute congestive œdema, for I wish to study especially the clinical point of view of the question. I will simply mention that it has successively been attributed to hyperacute asystolism, to spasm of the left ventricle, to vaso-motor trouble in the pulmonary system, to toxic action due to renal insufficiency (the affection being frequent in subjects suffering from arterio-sclerosis), to aortitis with peri-aortitis spreading to the neighbouring nervous plexi followed by vasomotor trouble in the lung.

It is especially in arterio-sclerosis and in patients suffering from aortic trouble, particularly aortic insufficiency of endarteric origin, to use the late Professor Peter's expression, that one observes this variety of pulmonary complication. It may also be noted in pregnant women suffering from mitral stenosis, and it then gives rise to symptoms which have been described in a masterly fashion by the same author. These symptoms may appear towards the end of the third month, more frequently towards the fifth month, and in some cases only towards the end of pregnancy. Their onset is generally sudden, sometimes "fulminating," and the prognosis is extremely grave. Immediate phlebotomy is necessary, and sometimes premature delivery has to be brought about. Death of the fœtus nearly always takes place.

### Pulmonary Causes.

Other causes may give rise to dyspnœa in cardiac patients. Foci of double broncho-pneumonia and infarcts, so well described by Laennec, may develop. They are more frequent in mitral lesions (stenosis or incompetency), with formation of blood clots in the right auricle and ventricle and subsequent embolism in the pulmonary artery or its branches. They may also give rise to foci of pulmonary hæmorrhage, which, whatever be their mode of production, produce sufficiently clear symptoms; one finds in a limited region of the lung slight dulness on percussion, diminution of the respiratory murmur, and mucous râles. If the infarct is very large it may give rise to tubular breathing. As regards functional trouble, dyspnœa is the capital and sometimes only symptom, but the diagnosis remains always a little uncertain until the patient begins to expectorate sputum of a dark brown, or quite black, colour, brought up in small rounded fragments.

In well-established heart disease an important cause of dyspnœa may be the existence of pleurisy with or without effusion. Pleurisy is very frequent in patients who die of heart affection, and at the post-mortem one finds pleural adhesions more or less extensive, and sometimes even pleural symphysis over the focus of congestion or pulmonary apoplexy. I have found effusion in quite a number of cases; as a rule it is moderate in quantity, a large quantity of liquid being rather rare. The fluid is serous, citrine, and only slightly coagulable. The inflammation affects more often the right side and occupies the general cavity of the pleura. The effusion, however, may be encysted, and in one of Rendu's cases it had collected between the diaphragm and the lung, the anterior and external side of which adhered intimately to the chest wall; posteriorly, the liquid extended right up to the right apex. In 1903 Dr. Renon published the account of a very interesting case, in which the liquid was purulent, and was situated between the base of the right lung and the diaphragm, and had collected below and *in front* of the chest wall. The onset of pleural effusion in heart patients is generally slow and insidious; sometimes its development is not due to a single cause. In some cases it is due to inflammation having attacked primarily the pericardium and then spread to the pleura; in other cases, especially in subjects suffering from arterio-sclerosis, it has been considered as following acute congestive œdema of the lung which, as we have seen, is so frequent in these cases.

### Pleurisy in Cardiac Cases.

Habitually, however, pleurisy in cardiac subjects is due to irritation of the serous membrane caused by some lesion of the subjacent pulmonary tissue. The lesions vary in nature with the different cases, but most commonly consist in pulmonary hæmorrhage. This explains why pleural effusion is more frequent in mitral affections. As regards diagnosis, there is no difficulty in recognising the presence of this complication, for the dulness on percussion, the diminution or total abolition of vocal fremitus, and of the respiratory murmur, the soft and distant bronchial "soufle," ægophony, etc., are characteristic. It is necessary, however, to distinguish pleurisy with effusion from simple hydrothorax. In pleurisy effusion is more frequent in the right side, and is nearly always unilateral; it generally gives rise to more or less dyspnœa even when the quantity of fluid is small. In hydrothorax the effusion is bilateral, it comes on in the later stages of heart disease and co-exists with œdema and dropsy in other regions. Cytological examination shows the presence of large endothelial cells either isolated or in groups, whilst in pleurisy the fluid is sero-fibrinous and contains numerous polynuclear leucocytes as well as endothelial cells. As one might expect, the presence of fluid in the pleura causes marked aggravation of the dyspnœa; to quote Gendrin's words, "when pleural effusion supervenes it is not rare to see heart patients—in whom up to that moment all the functions had been more or less regular—suddenly present marked aggravation of their state. The intensity of the dyspnœa in these cases is not in direct proportion to the quantity of fluid in the pleura, for that quantity is most often quite small; it is due to the fact that effusion comes on during the period of hyposystolism, and especially that of asystolism, when the slightest trouble in the pulmonary track gives rise to a great increase of the severity of the dyspnœa. If the effusion does not diminish rapidly by the exhibition of diuretics and purgatives, the dyspnœa may become so violent, that thoracentesis has to be performed immediately.

### Toxic Dyspnœa.

Toxic dyspnœa complicates mechanical dyspnœa (due in the majority of cases to passive congestion), when the kidneys are the seat of lesions which constitute "cardiac kidney." The disease from being simply cardiac becomes cardio-renal. The dyspnœa presents the characters of that due to renal insufficiency or to uræmia, with paroxysmal attacks and Cheyne Stokes respiration; it necessitates the same treatment.

One must not forget that in certain cardiac cases with arterio-sclerosis, Cheyne Stokes breathing may not be due to uræmia, but simply to cerebral ischæmia. In the production of the above symptoms one must not lose sight of the rôle of urinary toxicity. The latter presents habitually its normal characters when there is not any well marked hypertrophic dilatation or trouble of the peripheral circulation; but it increases notably, and may even attain double its normal intensity when there is hypertrophy. The examination of the urine is therefore very important in all heart cases, for it furnishes extremely useful information both as regards the nature of the dyspnœa and the therapeutical indications. The methylene blue test, the test for albumin, for urobilin, for biliary pigments and acids should never be omitted; estimation of urea, uric acid, phosphates and chlorides is very necessary. The estimation of chlorides is all the more important as we are now aware of the great rôle the retention of these salts plays in the pathogeny of œdema.

### Nervous and Reflex Cases.

Cardiac dyspnœa may, lastly, be of a nervous or reflex nature. Habitually in these cases the cause resides in dyspeptic trouble of a gastro-hepatic nature. These patients are not really cardiacs, but those in whom the heart trouble has, for starting point, affections of the stomach and liver which indirectly influence the heart. The primary digestive trouble gives rise to dyspnœic difficulty, which in its turn is followed by cardiac disturbance, producing the symptoms of dilatation of the right side of the heart, with even, in some cases, tricuspid insufficiency. I have not the time to go into details; I will only say that most frequently there is an actual thirst for air, rather than real dyspnœa; in a few exceptional cases, however, orthopnœa has been observed resembling the violent dyspnœa symptomatic of pulmonary embolism.

To sum up then, gastro-hepatic dyspepsia reacts on the right heart through the intermediary of the lung, and the reflex arc is constituted as follows: Nervous irritation, starting from the stomach or liver, excites the contractility of the pulmonary capillary vessels, which, as a consequence, gives rise to hypertrophic dilatation of the right ventricle; the latter causing, in extreme cases, tricuspid insufficiency. Reflex dyspnœa is not due to the non-penetration of air in the lung, but to the fact that the blood does not come into contact with the air in the alveoli on account of the spasmodic contraction of the pulmonary vessels.

